# Inhibition of the Keap1/Nrf2 Signaling Pathway Significantly Promotes the Progression of Type 1 Diabetes Mellitus

**DOI:** 10.1155/2021/7866720

**Published:** 2021-02-05

**Authors:** Yanmei Lou, Muyan Kong, Leyan Li, Yu Hu, Wenjun Zhai, Xiaoxiao Qi, Zhongqiu Liu, Jinjun Wu

**Affiliations:** Joint Laboratory for Translational Cancer Research of Chinese Medicine of the Ministry of Education of the People's Republic of China, International Institute for Translational Chinese Medicine, Guangzhou University of Chinese Medicine, Guangzhou, Guangdong 510006, China

## Abstract

Type 1 diabetes mellitus (T1DM) is an autoimmune disease characterized by insulin deficiency due to pancreatic *β*-cell damage and leads to hyperglycemia. The precise molecular mechanisms of the etiology of T1DM are not completely understood. Oxidative stress and the antioxidant status of pancreatic *β*-cells play a vital role in the pathogenesis and progression of T1DM. The Keap1/Nrf2 signaling pathway plays a critical role in cellular resistance to oxidative stress. This study is aimed at investigating the role of the Keap1/Nrf2 signaling pathway in the progression of T1DM. An alloxan- (ALX-) stimulated T1DM animal model in wild-type (WT) and Nrf2 knockout (Nrf2^−/−^) C57BL/6J mice and a mouse pancreatic *β*-cell line (MIN6) were established. Compared with the tolerant (ALX exposure, nondiabetic) WT mice, the sensitive (ALX exposure, diabetic) WT mice exhibited higher blood glucose levels and lower plasma insulin levels. The Keap1/Nrf2 signaling pathway was significantly inhibited in the sensitive WT mice, which was reflected by overexpression of Keap1 and low expression of Nrf2, accompanied by a marked decrease in the expression of the antioxidative enzymes. Compared with WT mice, the Nrf2^−/−^ mice had an increased incidence of T1DM and exhibited more severe pancreatic *β*-cell damage. The results of in vitro experiments showed that ALX significantly inhibited the viability and proliferation and promoted the apoptosis of MIN6 cells. ALX also markedly increased intracellular ROS production and caused DNA damage in MIN6 cells. In addition, the Keap1/Nrf2 signaling pathway was significantly inhibited in the damaged MIN6 cells. Moreover, Nrf2 silencing by transfection with Nrf2 siRNA markedly exacerbated ALX-induced MIN6 cell injury. Conclusively, this study demonstrates that inhibition of the Keap1/Nrf2 signaling pathway could significantly promote the incidence of T1DM. This study indicates that activation of Keap1/Nrf2 signaling in pancreatic *β*-cells may be a useful pharmacological strategy for the clinical prevention and treatment of T1DM.

## 1. Introduction

Type 1 diabetes mellitus (T1DM), which is a chronic autoimmune disease, is characterized by hyperglycemia resulting from insulin deficiency that occurs as a consequence of pancreatic islet *β*-cell damage [1, 2]. According to the International Diabetes Federation, the worldwide prevalence of diabetes in 2019 was 463 million and is predicted to reach 578 million by 2030 and 700 million by 2045 (https://diabetesatlas.org). T1DM accounts for approximately 10% of all diabetes worldwide. The incidence of T1DM is increasing by 3–4% annually, most notably in children and adolescents [3, 4]. Moreover, patients with T1DM experience substantial morbidity and mortality due to chronic complications [5]. Currently, patients with T1DM depend on lifelong insulin injections to maintain glucose levels as close to normal as possible to avoid hypoglycemia [2, 5]. Aside from insulin therapy, identifying novel targets to protect pancreatic islet *β*-cells from damage has become an attractive strategy for the prevention and/or treatment of T1DM.

It is widely accepted that both genetic and environmental factors are responsible for the pathogenesis and progression of T1DM [6]. However, the particular causes and precise pathogenic mechanisms of this disease are not completely understood. Growing evidence suggests that oxidative stress and the antioxidant status of pancreatic *β*-cells play a vital role in the development of T1DM [7–9]. Oxidative stress is an imbalance of reactive oxygen species (ROS) and antioxidants in the body, which can lead to cell and tissue damage, ultimately contributing to the development of a range of chronic conditions, such as cancer, diabetes, and heart disease [10]. Substantial evidence exists that oxidative stress can produce cytotoxicity to pancreatic *β*-cells, impair *β*-cell function, decrease insulin gene expression, and actively participate in the progression of T1DM [7–9]. Moreover, oxidative stress is responsible for the development of numerous T1DM complications, including neuropathy, retinopathy, nephropathy, and cardiovascular diseases [11, 12]. Hence, protecting pancreatic *β*-cells from oxidative stress-stimulated injury has been a broadly effective method for preventing the onset of T1DM.

Kelch-like ECH-associated protein 1/nuclear factor erythroid 2-related factor 2 (Nrf2) is the most important antioxidant signaling pathway that plays a critical role in cellular defenses against oxidative stress to counteract accumulating ROS in the body [13, 14]. Under unstressed conditions, Keap1 acts as a repressor, combines with Nrf2, and suppresses Nrf2 activity in the cytosol. Upon exposure to oxidative stress or pharmacological activation, Nrf2 rapidly dissociates from Keap1 and translocates into the nucleus, where it binds to antioxidant response elements in the promoter of target genes, thereby promoting the transcription of a wide array of antioxidative genes, mainly NAD(P)H: quinone oxidoreductase 1 (NQO1) and heme oxygenase-1 (HO-1) [13, 14]. The Keap1/Nrf2 signaling pathway has been proven to take part in a variety of diseases that commence with oxidative stress, including T1DM and the associated complications. For example, the protective functions of the Keap1/Nrf2 signaling pathway against T1DM have been studied in a streptozotocin-induced mouse model of T1DM [15, 16]. Nrf2 induction by treatment with Nrf2 activators could protect pancreatic *β*-cells from ROS-stimulated damage and effectively diminish endogenous oxidative stress to prevent the pathogenesis and progression of T1DM [15, 17, 18]. Keap1 deletion, which results in Nrf2 induction, could significantly repress the development of T1DM in nonobese diabetic mice [19]. Although evidence exists that activation of the Keap1/Nrf2 signaling pathway exerts favorable effects on T1DM, systematic in vivo and in vitro studies involving the intervention of the Keap1/Nrf2 signaling pathway on the onset of T1DM have not yet been explored. In addition, there is little information regarding whether and how the Keap1/Nrf2 signaling pathway contributes to the development of T1DM in Nrf2 knockout (Nrf2^−/−^) mice. Moreover, the different activation responses of the Keap1/Nrf2 signaling pathway between sensitive and tolerant mice exposed to ALX-induced T1DM are not yet completely understood.

To address these questions, an ALX-stimulated T1DM animal model in wild-type (WT) and Nrf2 knockout (Nrf2^−/−^) C57BL/6J mice and a mouse pancreatic *β*-cell line (MIN6) were established. These in vitro and in vivo experiments were designed to investigate the role of the Keap1/Nrf2 signaling pathway in the pathogenesis and progression of T1DM. The objectives of the present study were (1) to measure the modulation of the Keap1/Nrf2 signaling pathway between wild-type (WT) C57BL/6J sensitive and tolerant mice exposed to ALX; (2) to confirm the involvement of the Keap1/Nrf2 signaling pathway in the development of T1DM in Nrf2^−/−^ C57BL/6J mice; (3) to evaluate the impact of ALX on the growth, proliferation, apoptosis, and DNA damage of MIN6 cells, as well as on intracellular ROS production; (4) to evaluate the regulation of ALX on the Keap1/Nrf2 signaling pathway in MIN6 cells; and (5) to illuminate the role of the Keap1/Nrf2 signaling pathway in ALX-induced MIN6 cell injury by Nrf2 silencing. The results from the present study will expand our knowledge pertaining to the critical role of the Keap1/Nrf2 signaling pathway in T1DM, which might provide a useful pharmacological strategy for the clinical prevention and treatment of T1DM based on specifically targeting the Keap1/Nrf2 signaling pathway.

## 2. Materials and Methods

### 2.1. Chemicals and Reagents

The antibodies to Nrf2 and Keap1 were obtained from Abcam Corporation (Cambridge, UK), and HO-1, NQO1, and *β*-actin antibodies were purchased from Santa Cruz Biotechnology (Santa Cruz, CA), and phosphorylated H2A.X (p-H2A.X) antibody was got from Cell Signaling Technology Inc. (Boston, USA). Insulin and glucagon antibodies were purchased from Servicebio Co., Ltd. (Wuhan, China). Annexin V-FITC Apoptosis Detection Kit and Cellular Reactive Oxygen Species Detection Assay Kit were obtained from Thermo Fisher Scientific (Waltham, Massachusetts, USA). Besides, Mouse Insulin ELISA Kit and blood glucose test strips were, respectively, purchased from CUSABIO (Wuhan, China) and Roche (Basel, Switzerland). Nrf2-specific siRNA (siNrf2), control siRNA (siCon), and riboFECT™ CP transfection kit were purchased from RiboBio Co., Ltd. (Guangzhou, China). Alloxan (ALX) and insulin were purchased from Sigma-Aldrich (St. Louis, MO, USA) and Novo Nordisk (Denmark), respectively. Comet Assay Kit was bought from TREVIGEN, Inc. (Gaithersburg, Maryland). All other chemicals were of analytical reagent grade or better.

### 2.2. Cell Culture

A mouse pancreatic *β*-cell line (MIN6) was obtained from FuHeng Biology (Shanghai, China). MIN6 cells were cultured in RPMI 1640 medium containing 10% fetal bovine serum (FBS), 100 U/mL penicillin, and 0.1 mg/mL streptomycin. And the cells were routinely cultured in a constant incubator with 5% CO_2_ and 37°C saturation humidity.

### 2.3. Animals and Treatments

Animal experiment protocols were reviewed and approved by the Guangzhou University of Chinese Medicine Animal Care and Use Committee (Guangzhou, China) and conducted in accordance with the ethical standards and national guidelines. Male-specific pathogen-free wild-type (WT) C57BL/6 mice (4-6 weeks old, 18-22 g) were supplied from the Laboratory Animal Center of Sun Yat-Sen University, Guangzhou, China. Male Nrf2^−/−^ C57BL/6 mice weighing 18-22 g were supplied by the Riken Bioresource Center (Koyadai, Japan). The genotypes of Nrf2^−/−^ C57BL/6 mice were validated by PCR and gene sequencing according to our previous study [20, 21]. All mice were bred under a controlled temperature of 23 -25°C with a 12-hour day/night cycle. Twenty WT and sixteen Nrf2^−/−^ C57BL/6 mice were injected with ALX (60 mg/kg body weight) via the tail vein after 24 hours of fasting. Seventy-two hours later, the tail blood was taken, and the first fasting blood glucose (FBG) was measured using an ACCU-CHEK blood monitor (Roche, Basel, Switzerland) and test strips. Then, the FBG was measured, and the body weights were recorded every three days for one month. At the end of the treatment, the mice were sacrificed, and the organs were removed and weighed. The organ indexes (organ weight/body weight) were statistically analyzed. According to the FBG values [22, 23], the mice were divided into three groups as follows: control group (without ALX exposure), tolerant group (ALX exposure, nondiabetic mice with an FBG < 11.1 mmol/L), and sensitive group (ALX exposure, diabetic mice with an FBG ≥ 11.1 mmol/L).

### 2.4. Hematoxylin and Eosin (H&E) Staining

At the end of treatment, the pancreatic tissues of the mice were removed, fixed with 4% paraformaldehyde for over 24 hours, and then embedded in paraffin. The slices were prepared 4 *μ*m for hematoxylin-eosin (HE) staining and were photographed under a light microscope magnification of 400x.

### 2.5. Immunohistochemistry

After processing as mentioned above, the pancreatic sections (4 *μ*m thick) were deparaffinized, rehydrated, and incubated with sodium citrate for antigen retrieval. Next, the sections were incubated in primary anti-insulin (1 : 500) antibody at 4°C overnight. Following that, a secondary antibody was applied for 30 min at room temperature. Sections were processed with SABC and developed with 3,3-diaminobenzidine (DAB). The quantitative analysis of insulin expression was performed by using ImageJ software.

### 2.6. Enzyme-Linked Immunosorbent Assay (ELISA)

Blood samples were collected before the mice were sacrificed. The insulin concentrations in mouse plasma were assessed by using a Mouse Insulin ELISA Kit according to the manufacturer's instructions. And the absorbance was read at 450 nm using a Victor X3 microplate reader.

### 2.7. MTT Assay

An MTT assay was carried out to examine the effects of ALX on the MIN6 cell viability. MIN6 cells were seeded in 96-well plates at a density of 1 × 10^4^ cells/mL. After incubation for 24 h, the cells were treated with various concentrations of ALX (0, 0.75, 1.5, 3, 6, and 12 mM) and continued to be incubated at 37°C for another 6 h. At the end of the incubation, the cells were incubated with 200 *μ*L MTT (0.5 mg/mL) for additional 4 h at 37°C. Then, supernatant was discarded; 150 *μ*L of DMSO was added to each well and incubated for 10 min. Finally, the absorbance of each well was measured at 490/570 nm using a microplate reader. Cell viability was expressed as a percentage of the vehicle control.

### 2.8. EdU Assay

Proliferation of MIN6 cells was verified by EdU assay based on the protocols. MIN6 cells were seeded into 96-well plates and treated with various concentrations of ALX (0, 1.5, 3, and 6 mM) for 6 h. Then, the cells were treated with 50 *μ*M of EdU for 2 h at 37°C. Afterwards, the cells were fixed in 4% formaldehyde, incubated with glycine, and then incubated with 1x Apollo 488 working solution, permeabilized with Triton X-100, and finally stained with Hoechst 33342 dye. Subsequently, the cell nuclei were stained with Hoechst 33342 and visualized under a fluorescence microscope (Leica, Germany). The cell proliferation index was determined as the ratio of EdU to Hoechst 33342 and calculated based on the green color of positive cells.

### 2.9. Apoptosis Assay

Cell apoptosis was detected by Annexin V-FITC/propidium iodide (PI) staining according to the manufacturer's instructions. Briefly, MIN6 cells were seeded in six-well plates and treated with ALX (0, 1.5, 3, and 6 mM) for 6 h. Then, the cells were washed with cold PBS twice and stained with Annexin V-FITC and PI in binding buffer for 15 min at room temperature in the dark. Stained cells were quantified by flow cytometry (BD Biosciences, San Jose, CA, USA). The apoptosis rate of cells was analyzed using FlowJo 7.6.1 software (Tree Star, Inc., Ashland, OR, USA).

### 2.10. Comet Assay

A Comet Assay Kit was used to quantitate DNA damage according to the manufacturer's instructions. In short, MIN6 cells were treated with ALX (0, 1.5, 3, and 6 mM) for 6 h. After incubation, the cells were harvested. The following detailed procedures were shown as previously described [13]. Images were captured by using a Leica 3000B fluorescence microscope (Leica, Germany).

### 2.11. Intracellular ROS Measurement

MIN6 cells were seeded in six-well plates and exposed to ALX (0, 1.5, 3, and 6 mM) for 6 h. N-Acetyl-L-cysteine (NAC, 2 mM), a well-known ROS inhibitor, was used as a positive control. At the end of incubation, the cells were incubated with CM-H2DCFDA (5 *μ*M) at 37°C for 30 min. The fluorescence signals were detected by flow cytometry (BD Biosciences, San Diego, CA, USA) and a confocal microscope (Leica, Germany), respectively. The results were analyzed with FlowJo 7.6 software. The results were expressed as the fold change of the fluorescence intensity over the control.

### 2.12. Western Blot Analysis

At the end of treatment, both mouse pancreatic tissues and MIN6 cells were lysed with RIPA buffer supplemented with a protease inhibitor cocktail. The nuclear protein extracts of MIN6 were also prepared using NE-PER nuclear and cytoplasmic extraction reagents (Rockford, IL, USA) according to the manufacturer's instructions. Protein concentrations were determined with a BCA estimation kit according to the manufacturer's instructions. Western blotting was performed as previously described [24] using primary antibodies against Keap1, Nrf2, HO-1, NQO1, and *β*-actin (1 : 2000). Densitometry of the band intensities was determined using ImageJ software.

### 2.13. Immunofluorescence

For immunofluorescence staining for cells, MIN6 cells were seeded on confocal dishes and exposed to ALX (0, 1.5, 3, and 6 mM) for 6 h. At the end of the incubation, the cells were fixed in paraformaldehyde, permeabilized with TritonX-100, and blocked with bovine serum albumin. Then, the cells were incubated with a p-H2A.X (1 : 100) or a Nrf2 (1 : 200) antibody at 4°C overnight and then stained with a secondary fluorescent antibody (1 : 200; Alexa Fluor 568, Abcam Inc., Cambridge, MA, USA). Finally, the cells were incubated with DAPI for another 20 min. For immunofluorescence staining for mouse pancreatic tissues, pancreatic sections were stained with an insulin (1 : 200) or an anti-glucagon (1 : 200) antibody and then stained with a secondary fluorescent antibody. Fluorescence signals were detected using a Leica TCS SP8 confocal fluorescence microscope (Leica, Germany). The relative fluorescence of p-H2A.X and Nrf2 in the cells and the insulin- and glucagon-positive areas of pancreatic sections were analyzed by ImageJ software.

### 2.14. siRNA Interference

Nrf2-targeting siRNA (siNrf2, 50 nM) or control siRNA (siCon) were transfected into the MIN6 cells for 24 h, respectively, using the RiboFECT™ CP transfection kit according to the manufacturer's instructions. After the transfection, the cells were exposed to ALX (3 mM) for 6 h. At the end of treatment, the cells were collected for MTT assay, EdU assay, apoptosis assay, comet assay, immunofluorescence, or ROS measurement, respectively, under the same conditions described above.

### 2.15. Data Analysis

Data are presented as mean ± standard deviation (SD). One-way ANOVA was used for comparison between different groups. The significance of incidence of T1DM was analyzed using the *χ*^2^ test, and correlation analyses were performed using the Pearson product–moment correlation by SPSS 19.0. All statistical analysis was performed using SPSS 19.0. Differences were considered significant at *p* < 0.05.

## 3. Results

### 3.1. The Susceptibility to ALX-Induced T1DM Varies Significantly among Different Individuals of WT C57BL/6J Mice

**Figure 1:**
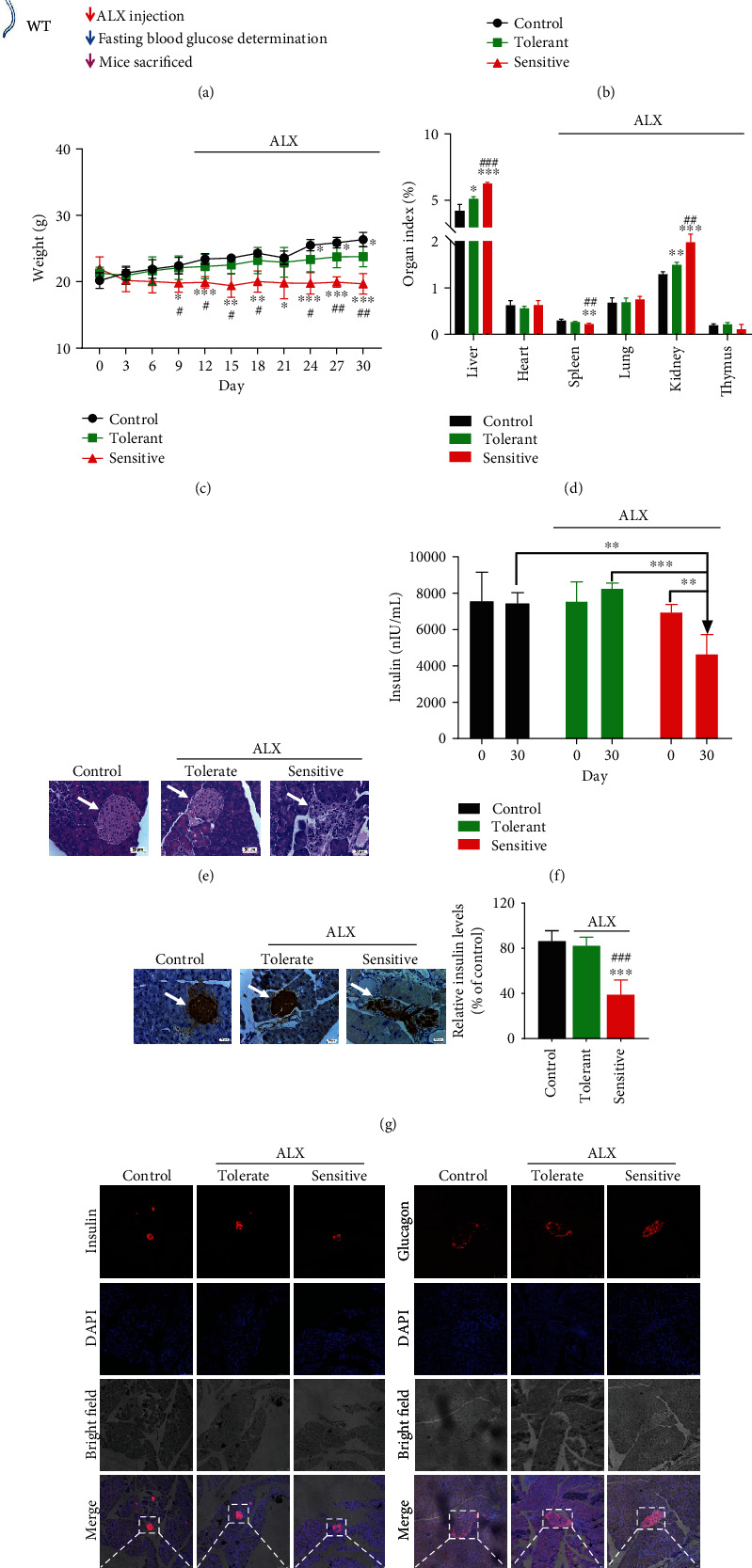
The susceptibility to ALX-induced T1DM varies significantly among different individuals of WT C57BL/6J mice. (a) A stimulated T1DM animal model in WT C57BL/6J mice was established by injection with ALX (60 mg/kg body weight) via the tail vein after 24 hours of fasting. The fasting blood glucose concentrations and body weights were measured every three days for one month. (b) Average blood glucose concentration curves for the mice. (c) Average body weight curves for the mice. (d) Organ indexes for mouse livers, hearts, spleens, lungs, kidneys, and thymuses. (e) Representative images showing hematoxylin and eosin staining of pancreatic islets for the mice. (f) The insulin concentrations in mouse plasma were assessed by using an ELISA Kit. (g) The insulin in the pancreatic islets of the mice was detected by immunohistochemistry. (h) The insulin and glucagon in the pancreatic islets of the mice were detected by immunofluorescence. The data represent the mean ± SD (*n* = 5). ^∗^*p* < 0.05, ^∗∗^*p* < 0.01, and ^∗∗∗^*p* < 0.001 compared with the control group; ^#^*p* < 0.05, ^##^*p* < 0.01, and ^###^*p* < 0.001 compared with the tolerant group.

As shown in Figure 1(a), a stimulated T1DM animal model in WT C57BL/6J mice was established by injection with ALX via the tail vein. The fasting blood glucose concentrations were measured every three days for one month. Interestingly, the blood glucose concentrations varied significantly among different individuals in mice exposed to the same ALX treatment. Among the twenty mice exposed to ALX, five mice were tolerant of ALX and were nondiabetic mice; fifteen mice were sensitive to ALX and were diabetic mice. Accordingly, the mice were divided into three groups: control group (without ALX exposure), tolerant group (ALX exposure, nondiabetic mice), and sensitive group (ALX exposure, diabetic mice). The blood glucose levels and plasma insulin levels were comparable in the control and tolerant mice. Compared with the tolerant mice, the sensitive mice exhibited higher blood glucose levels (Figure 1(b)). The body weights of the sensitive mice were significantly reduced with respect to the control and tolerant mice (Figure 1(c)). Changes in the organ indexes, including the liver, spleen, and kidney, among the mice were also observed (Figure 1(d)). Histological analyses of pancreatic islets showed more substantial islet mass reduction and islet degeneration, as well as more extensive inflammatory infiltration in the pancreatic islets in the sensitive mice compared with those in the tolerant mice (Figure 1(e)). Moreover, ELISA and IHC analyses showed that all the sensitive mice displayed lower plasma insulin levels than the control and tolerant mice (Figures 1(f) and 1(g)). In addition, immunofluorescent staining showed fewer insulin- and more glucagon-positive cells in the pancreatic sections of the sensitive mice compared with those in the tolerant mice (Figure 1(h)).

### 3.2. The Keap1/Nrf2 Signaling Pathway Was Significantly Inhibited in the Sensitive Mice

**Figure 2:**
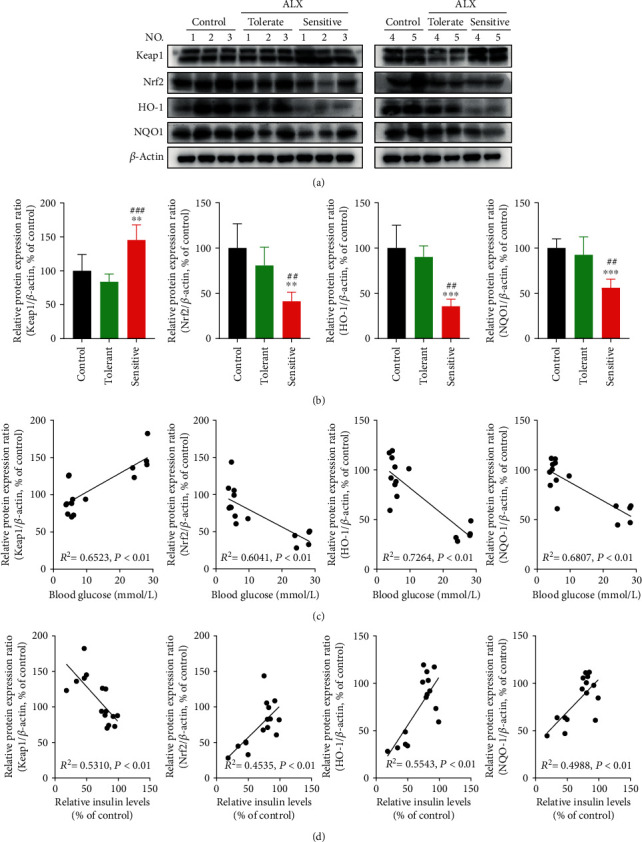
The Keap1/Nrf2 signaling pathway was significantly inhibited in the sensitive mice. (a, b) The protein levels of Keap1, Nrf2, HO-1, and NQO1 in the pancreatic islets of the mice were detected by Western blot analysis. (c) Pairwise correlation between blood glucose concentrations and protein levels of Keap1, Nrf2, HO-1, and NQO1. (d) Pairwise correlation between insulin levels and protein levels of Keap1, Nrf2, HO-1, and NQO1. The correlations were analyzed by using Pearson analysis. The data represent the mean ± SD (*n* = 5). ^∗∗^*p* < 0.01 and ^∗∗∗^*p* < 0.001 compared with the control group; ^##^*p* < 0.01 and ^###^*p* < 0.001 compared with the tolerant group.

To investigate the role of the Keap1/Nrf2 signaling pathway in the pathogenesis and progression of T1DM, the protein levels of Keap1 and Nrf2, as well as two target antioxidative genes HO-1 and NQO1, were measured in the control, tolerant, and sensitive mice, respectively. As shown in Figures 2(a) and 2(b), the sensitive mice exhibited significantly higher Keap1 expression and lower Nrf2 expression (*p* < 0.01 or *p* < 0.001), as well as lower expression of HO-1 and NQO1 than the control and tolerant mice (*p* < 0.01 or *p* < 0.001). Pearson correlation analysis showed that the increased blood glucose concentrations were positively related to the higher Keap1 expression (Figure 2(c), *p* < 0.01) and were negatively related to the lower Nrf2, HO-1, and NQO1 expression (Figure 2(c), *p* < 0.01). Conversely, a strong negative correlation was also observed between the decreased insulin levels and higher Keap1 expression (Figure 2(d), *p* < 0.01); a significant positive correlation was also observed between the decreased insulin levels and lower Nrf2, HO-1, and NQO1 expression (Figure 2(d), *p* < 0.01).

### 3.3. The Keap1/Nrf2 Signaling Pathway Mediates the Pathogenesis and Progression of T1DM in Mice

**Figure 3:**
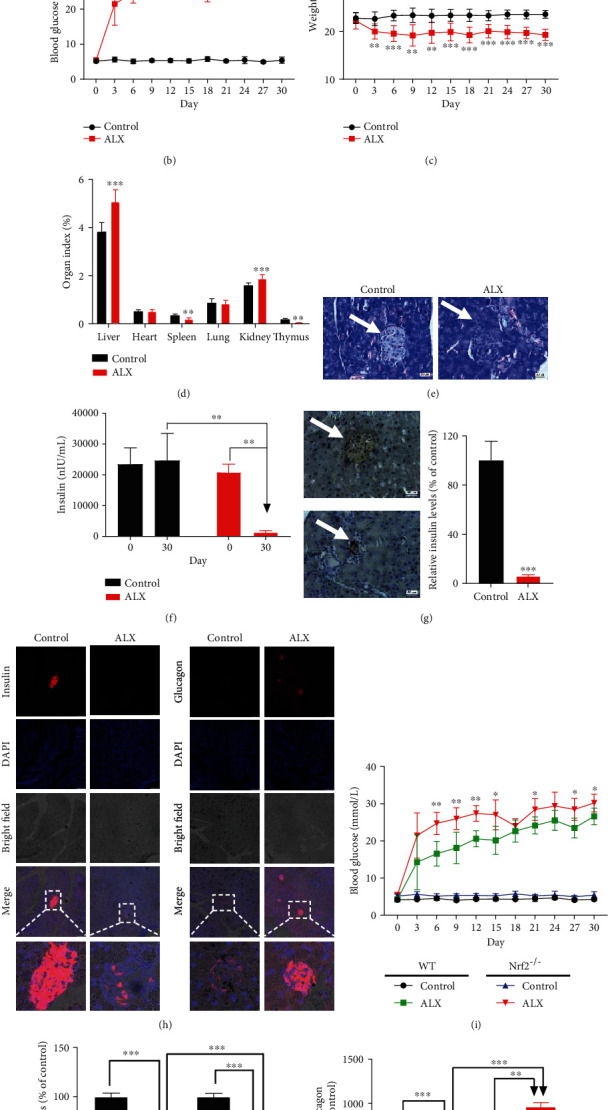
The Keap1/Nrf2 signaling pathway mediates the pathogenesis and progression of T1DM in mice. (a) A stimulated T1DM animal model in Nrf2^−/−^ C57BL/6J mice was established by injection with ALX (60 mg/kg body weight) via the tail vein after 24 hours of fasting. The fasting blood glucose concentrations and body weights were measured every three days for one month. (b) Average blood glucose concentration curves for the mice. (c) Average body weight curves for the mice. (d) Organ indexes for mouse livers, hearts, spleens, lungs, kidneys, and thymuses. (e) Representative images showing hematoxylin and eosin staining of pancreatic islets for the mice. (f) The insulin concentrations in mouse plasma were assessed by using an ELISA Kit. (g) The insulin in the pancreatic islets of the mice was detected by immunohistochemistry. (h) The insulin and glucagon in the pancreatic islets of the mice were detected by immunofluorescence. Comparison of the blood glucose concentrations (i), insulin (j), and glucose levels (k) between the WT and Nrf2^−/−^ mice exposed to the same ALX treatment. The data represent the mean ± SD (*n* = 5). ^∗∗^*p* < 0.01 and ^∗∗∗^*p* < 0.001 compared with the control group for (b)–(g); ^∗∗∗^*p* < 0.001 compared with the WT group for (i)–(k).

To further confirm the involvement of the Keap1/Nrf2 signaling pathway in the progression of T1DM, an ALX-stimulated T1DM animal model in C57BL/6J background Nrf2^−/−^ mice was established (Figure 3(a)). All the Nrf2^−/−^ mice exposed to ALX showed significantly higher blood glucose levels (Figure 3(b), *p* < 0.01 or *p* < 0.001) and lower body weights (Figure 3(c), *p* < 0.01 or *p* < 0.001) compared with those in the control mice. Significant changes in the organ indexes, including the liver, spleen, kidney, and thymus, among the mice were also observed (Figure 3(d), *p* < 0.01 or *p* < 0.001). More substantial islet mass reduction and islet degeneration and more extensive inflammatory infiltration in the pancreatic islets were observed in the ALX-treated Nrf2^−/−^ mice compared with those in the control mice (Figure 3(e)). Moreover, the Nrf2^−/−^ mice displayed lower plasma insulin levels than the control mice (Figures 3(f) and 3(g), *p* < 0.01 or *p* < 0.001). In addition, the Nrf2^−/−^ mice showed fewer insulin- and more glucagon-positive cells in the pancreatic sections compared with those in the control mice (Figure 3(h)).

In contrast to the WT mice under the same ALX exposure, Nrf2^−/−^ mice exhibited a significantly increased incidence of T1DM (Table 1, *p* < 0.001). Higher blood glucose levels were detected in the Nrf2^−/−^ mice during the experiments (Figure 3(i), *p* < 0.05 or *p* < 0.01). Moreover, the Nrf2^−/−^ mice displayed less insulin- (Figure 3(j), *p* < 0.001) and more glucagon-positive cells (Figure 3(k), *p* < 0.001) in the pancreatic sections compared with those in the WT mice.

### 3.4. ALX Significantly Induced Injury toward Pancreatic *β*-Cells

**Figure 4:**
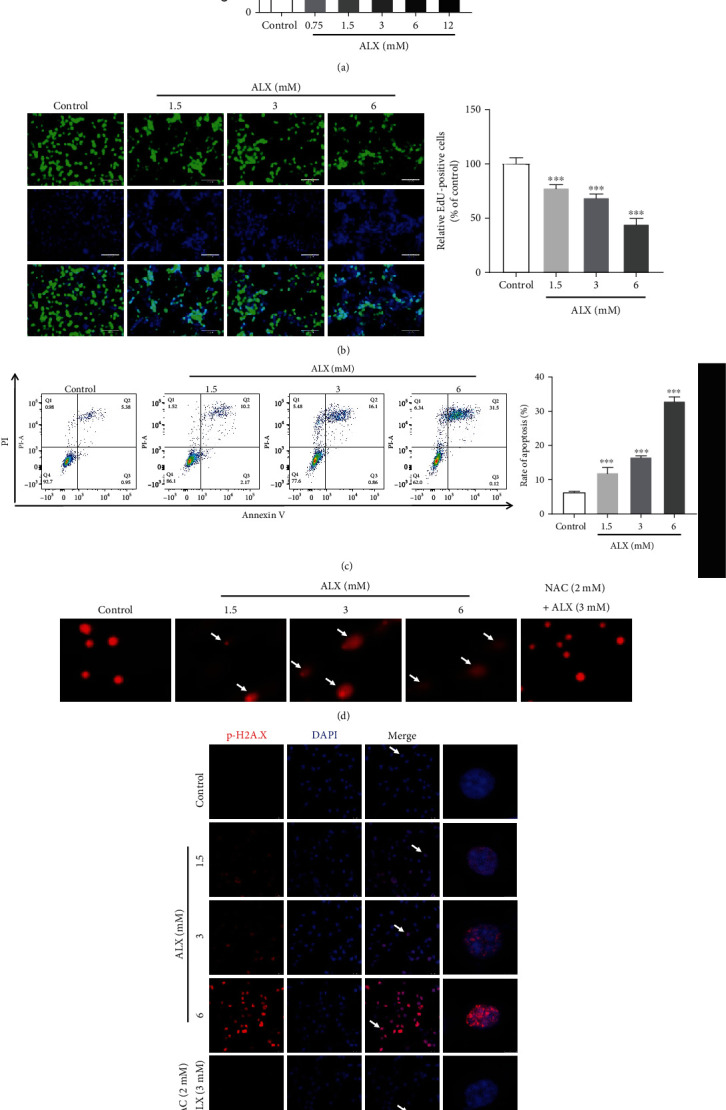
ALX significantly induced injury toward pancreatic *β*-cells. (a) The cytotoxicity of ALX (0.75 to 12 mM, 24 h) toward MIN6 cells was evaluated by using an MTT assay. (b) EdU incorporation was evaluated after treatment of cells with vehicle and ALX (1.5, 3, and 6 mM) for 24 h. The EdU-positive cells in each group were quantified as the percentage of those in the control group. (c) The impact of vehicle and ALX (1.5, 3, and 6 mM, 24 h) on MIN6 cell apoptosis was detected by Annexin V-FITC/propidium iodide (PI) staining. (d) Representative comet tail images showing the DNA damage response after treatment with vehicle and ALX (1.5, 3, and 6 mM) for 24 h. (e) Representative confocal images (scale bar: 50 *μ*m) of double-stained cells subjected to the same treatments and stained for p-H2A.X (red) and with DAPI (blue). The data represent the mean ± SD (*n* = 3). ^∗∗^*p* < 0.01 and ^∗∗∗^*p* < 0.001 compared with the control group.

To further determine how the Keap1/Nrf2 signaling pathway regulates the progression of T1DM, an ALX-stimulated injury of mouse pancreatic *β*-cell line (MIN6) was established. As shown in Figure 4(a), in contrast to the control MIN6 cells, ALX treatment at 0.75-12 mM for 24 h significantly decreased the cell viability in a dose-dependent manner (*p* < 0.01 or *p* < 0.001). ALX also markedly decreased the cell proliferation (Figure 4(b), *p* < 0.001), as well as increased the cell apoptosis (Figure 4(c), *p* < 0.001). The results from the comet assay showed that ALX could markedly enhance the fluorescence in migrated DNA and the tails of disrupted DNA fragments, suggesting that ALX could notably cause DNA damage of MIN6 cell (Figure 4(d)). N-Acetyl-L-cysteine (NAC), a well-known ROS inhibitor, could markedly reduce the ALX-induced long tails of disrupted DNA fragments. In addition, immunofluorescent staining showed higher expression of p-H2A.X, which is a verified marker for DNA double-strand breaks and could be used to evaluate the DNA damage, in the MIN6 cells exposed to ALX compared with that in the control cells (Figure 4(e)). NAC could significantly decrease the ALX-induced high expression of p-H2A.X.

### 3.5. ALX Significantly Promoted the Intracellular ROS Overproduction in Pancreatic *β*-Cells

**Figure 5:**
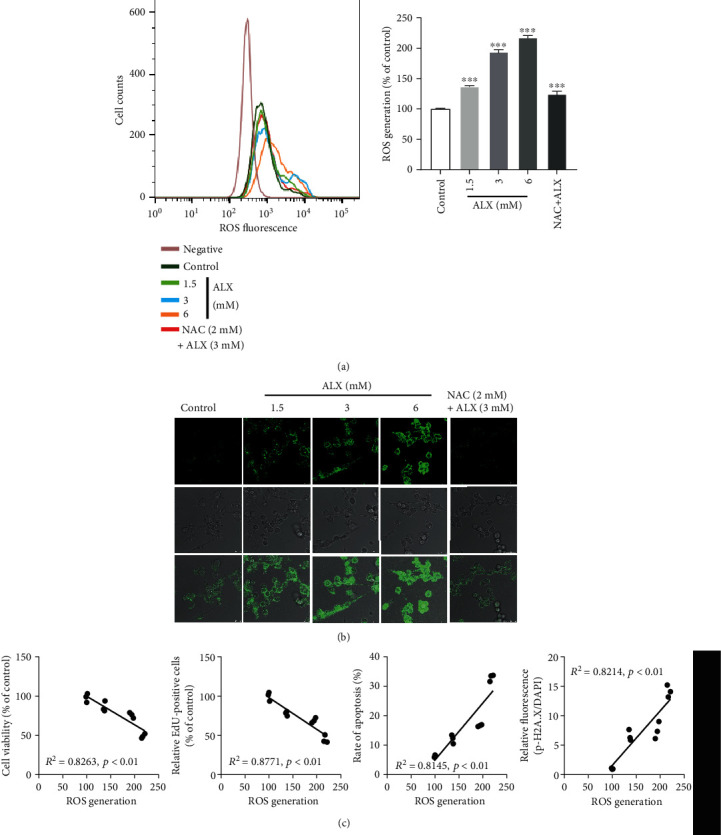
ALX significantly promoted intracellular ROS overproduction in pancreatic *β*-cells. (a) Intracellular ROS levels were detected by flow cytometry after exposure to vehicle and ALX (1.5, 3, and 6 mM) for 24 h. (b) Intracellular ROS generation was detected by fluorescence microscopy (scale bar: 50 *μ*m) after treatment with vehicle and ALX (1.5, 3, and 6 mM) for 24 h. (c) Pairwise correlation between ROS generation and cell viability, proliferation, cell apoptosis, or DNA damage after treatment with vehicle and ALX (1.5, 3, and 6 mM) for 24 h, respectively. The correlations were analyzed by using Pearson analysis. NAC, a well-known ROS inhibitor, was used as a positive control. The data represent the mean ± SD (*n* = 3). ^∗∗∗^*p* < 0.001 compared with the control group.

Intracellular ROS levels were detected by flow cytometry after exposure to ALX. As shown in Figure 5(a), ALX-treated MIN6 cells exhibited significantly higher ROS production compared with that in the control cells (*p* < 0.001). N-Acetyl-L-cysteine (NAC), a well-known ROS inhibitor, could markedly reverse the ALX-induced p-H2A.X overexpression (Figure 5(a), *p* < 0.001). Intracellular ROS generation was also detected by fluorescence microscopy and confirmed the results that ALX treatment could dose-dependently promote the intracellular ROS overproduction (Figure 5(b)). Pearson correlation analysis showed that the increased ROS production was negatively related to the decreased cell viability and proliferation (Figure 5(c), *p* < 0.01). Conversely, a strong positive correlation was observed between the ROS overproduction and increased cell apoptosis and DNA damage (Figure 5(c), *p* < 0.01).

### 3.6. ALX Significantly Inhibited the Keap1/Nrf2 Signaling Pathway in Pancreatic *β*-Cells

**Figure 6:**
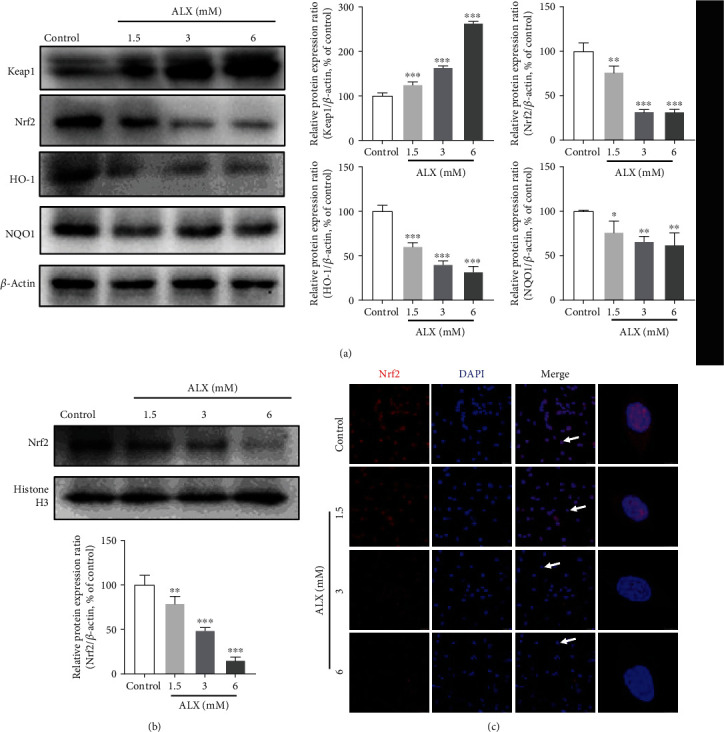
ALX significantly inhibited the Keap1/Nrf2 signaling pathway in pancreatic *β*-cells. (a) The protein levels of Keap1, Nrf2, HO-1, and NQO1 in whole-cell lysates after treatment with vehicle and ALX (1.5, 3, and 6 mM) for 24 h were detected by Western blot analysis. (b) The nuclear Nrf2 protein levels were also detected after the same treatment. (c) Representative confocal images (scale bar: 50 *μ*m) of MIN6 cells subjected to the same treatments and stained for Nrf2 (red) and with DAPI (blue). The data represent the mean ± SD (*n* = 3). ^∗^*p* < 0.05, ^∗∗^*p* < 0.01, and ^∗∗∗^*p* < 0.001 compared with the control group.

The impact of ALX on the Keap1/Nrf2 signaling pathway was detected by Western blot analysis. As shown in Figure 6(a), in contrast to the control MIN6 cells, ALX treatment at 1.5, 3, and 6 mM for 24 h significantly upregulated the Keap1 protein levels in whole-cell lysates in a dose-dependent manner (*p* < 0.001). The MIN6 cells exposed to ALX under the same conditions also exhibited a significant decrease in the protein levels of Nrf2, HO-1, and NQO1 compared with that in the control cells (Figure 6(a), *p* < 0.05, *p* < 0.01, or *p* < 0.001). The translocation of Nrf2 from the cytoplasm to the nucleus was further determined. It was observed that ALX treatment also markedly decreased the nuclear Nrf2 protein levels in a dose-dependent manner (Figure 6(b), *p* < 0.01 or *p* < 0.001). Moreover, immunofluorescent staining showed notably lower expression of Nrf2 in the MIN6 cells exposed to ALX compared with that in the control cells (Figure 6(c)).

### 3.7. The Keap1/Nrf2 Signaling Pathway Mediates ALX-Induced Pancreatic *β*-Cell Injury

**Figure 7:**
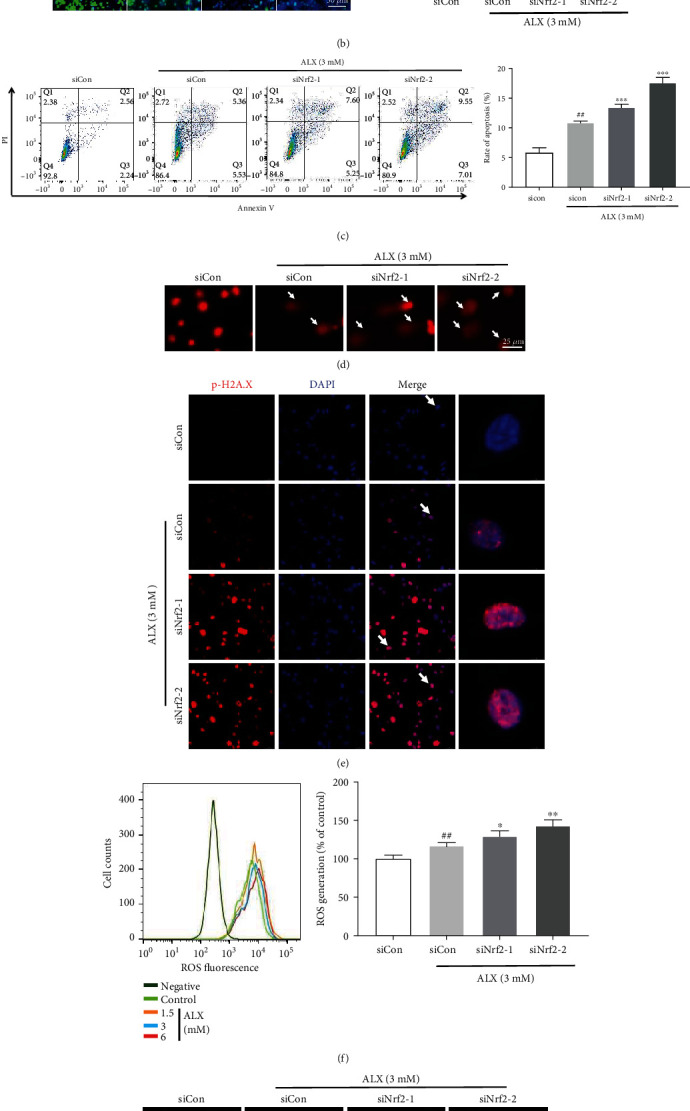
The Keap1/Nrf2 signaling pathway mediates ALX-induced pancreatic *β*-cell injury. The MIN6 cells were treated with vehicle and ALX (3 mM) in the presence or absence of Nrf2-specific siRNAs (siNrf2-1 or siNrf2-2); the control cells were only transfected with control siRNA (siCon). (a) Cell viability was determined by using an MTT assay. (b) Proliferation of MIN6 cells was verified by EdU assay. (c) The MIN6 cell apoptosis was detected by Annexin V-FITC/propidium iodide (PI) staining. (d) Representative comet tail images showing the DNA damage. (e) Representative confocal images (scale bar: 50 *μ*m) of MIN6 cells stained for p-H2A.X (red) and with DAPI (blue). (f) Intracellular ROS levels were detected by flow cytometry. (g) Intracellular ROS generation was detected by fluorescence microscopy (scale bar: 50 *μ*m). The data represent the mean ± SD (*n* = 3). ^##^*p* < 0.01 and ^###^*p* < 0.001 compared with the siCon group. ^∗^*p* < 0.05, ^∗∗^*p* < 0.01, and ^∗∗∗^*p* < 0.001 compared with the siCon plus ALX group.

To confirm the role of the Keap1/Nrf2 signaling pathway in ALX-stimulated injured MIN6 cells, Nrf2 expression was silenced in MIN6 cells by transfection with two Nrf2-specific siRNAs (siNrf2-1 or siNrf2-2). Cells transfected with siNrf2 showed significant reductions in Nrf2 expression compared to cells transfected with control siRNA (siCon) (Supplementary Fig. 1, *p* < 0.001). Compared with cells transfected with siCon and exposed to ALX, cells exposed to ALX and transfected with siNrf2-1 or siNrf2-2 exhibited significantly lower cell viability (Figure 7(a), *p* < 0.001) and proliferation (Figure 7(b), *p* < 0.001). In contrast to transfection with siCon, transfection with siNrf2-1 or siNrf2-2 also markedly increased the ALX-induced apoptosis of MIN6 cells (Figure 7(c), *p* < 0.001). In addition, the ALX-stimulated DNA damage of MIN6 cells could be markedly enhanced by transfection with siNrf2-1 or siNrf2-2 (Figures 7(d) and 7(e)). Furthermore, transfection with siNrf2-1 or siNrf2-2 strikingly promoted ALX-induced intracellular ROS overproduction with respect to transfection with siCon (Figures 7(f) and 7(g), *p* < 0.05 or *p* < 0.01).

## 4. Discussion

The precise molecular mechanisms of the pathogenesis and progression of T1DM are not completely understood. Evidence exists that oxidative stress can impair *β*-cell function and actively participate in the onset of T1DM. Keap1/Nrf2 is the most important antioxidant signaling pathway that plays a critical role in cellular defenses against oxidative stress. However, the role of the Keap1/Nrf2 signaling pathway in T1DM is not yet completely understood. Hence, in the present study, systematic in vitro and in vivo experiments were designed to investigate the involvement of the Keap1/Nrf2 signaling pathway in the development and progression of T1DM.

First, an ALX-stimulated T1DM animal model in WT C57BL/6J mice was established. The alloxan-induced mouse model of T1DM is a classic method for investigating the pathogenic mechanisms of this disease and exploring the therapeutic strategy against T1DM [25–27]. Interestingly, the susceptibility to ALX-induced T1DM varies significantly among different individual WT C57BL/6J mice exposed to the same ALX treatment. The body weights, blood glucose levels, and plasma insulin levels were comparable in the control and T1DM-tolerant mice. Compared with the T1DM-tolerant mice, the T1DM-sensitive mice exhibited higher blood glucose levels, lower plasma insulin levels, and lower body weights. In addition, the sensitive mice showed more substantial islet mass reduction and islet degeneration, as well as more extensive inflammatory infiltration in the pancreatic islets compared with those in the tolerant mice (Figure 1). It is well recognized that both genetic and environmental factors are responsible for the development and progression of T1DM [2, 6]. Thus, these results indicated that one or more potential target(s) might be involved in the delayed progression of T1DM among the tolerant mice through the protection of pancreatic *β*-cell function.

Growing evidence suggests that oxidative stress-induced damage to pancreatic *β*-cells is one of the key factors that contribute to the onset of T1DM [7–9]. Thus, identifying novel targets to protect pancreatic islet *β*-cells from oxidative stress-stimulated injury has become an attractive strategy for the prevention and treatment of T1DM. The Keap1/Nrf2 signaling pathway is the most critical regulator involved in cellular defenses against oxidative stress [13, 14], thereby potentially playing a vital role in the progression of T1DM. T1DM-sensitive mice exhibited higher Keap1 expression and lower Nrf2 expression, as well as lower expression of two target antioxidative genes, HO-1 and NQO1 (Figure 2). Correlation analysis showed that the increased blood glucose concentrations were positively related to higher Keap1 expression and were negatively related to lower Nrf2, HO-1, and NQO1 expression. Conversely, a strong negative correlation was observed between the decreased insulin levels and higher Keap1 expression, and a significant positive correlation was also observed between the decreased insulin levels and lower Nrf2, HO-1, and NQO1 expression (Figure 2). These findings revealed that the Keap1/Nrf2 signaling pathway was significantly inhibited in T1DM-sensitive mice and suggested that inhibition of the Keap1/Nrf2 signaling pathway could be tightly associated with the progression of T1DM. To confirm this hypothesis, C57BL/6J background Nrf2^−/−^ mice were established, which is a useful model for assessing the roles of Nrf2 in the development and progression of T1DM. Nrf2^−/−^ mice exhibited a significantly increased incidence of T1DM compared with WT mice under the same ALX exposure (Table 1). Higher blood glucose levels and lower plasma insulin levels were detected in the mice with Nrf2 deletion. Moreover, the Nrf2^−/−^ mice displayed more islet mass reduction and islet degeneration, as well as extensive inflammatory infiltration in the pancreatic islets compared with those in the WT mice (Figure 3). Taken together, these results from the in vivo studies demonstrated that the Keap1/Nrf2 signaling pathway participates in the pathogenesis and progression of T1DM.

To further determine how the Keap1/Nrf2 signaling pathway contributes to the prevention of T1DM, an ALX-stimulated injury mouse pancreatic *β*-cell line (MIN6) was established. In contrast to the control MIN6 cells, exposure to ALX significantly decreased cell viability and proliferation and increased cell apoptosis (Figure 4). These data suggested that ALX could significantly cause injury toward pancreatic *β*-cells. DNA damage has been confirmed to be one of the primary causes of pancreatic *β*-cell injury and thus could be tightly associated with increased T1DM risk [28, 29]. Hence, the impact of ALX on DNA damage was further investigated. The results from the comet assay showed that ALX could markedly enhance the fluorescence in migrated DNA and the tails of disrupted DNA fragments (Figure 4), suggesting that ALX could notably cause DNA damage in MIN6 cells. In addition, the expression of p-H2A.X, which is a verified marker for DNA double-strand breaks and could be used to evaluate DNA damage [30, 31], was determined. Exposure to ALX significantly resulted in the overexpression of p-H2A.X in the MIN6 cells (Figure 4). These results indicated that ALX could significantly induce DNA damage, which suggested that ALX could induce DNA damage, thereby causing pancreatic *β*-cell injury and finally promoting the onset of T1DM.

Oxidative stress has been proven to be one of the most potent factors that can lead to DNA damage [13, 32]. Therefore, the impact of ALX on intracellular ROS levels was further investigated. ALX significantly promoted intracellular ROS overproduction in MIN6 cells (Figure 5). Correlation analysis showed that the increase in ROS levels was closely and positively related to the promotion of ALX-induced DNA damage and pancreatic *β*-cell injury (Figure 5). It was speculated that ALX could promote intracellular ROS overproduction to cause DNA damage, thereby inducing toxicity toward MIN6 cells.

The Keap1/Nrf2 signaling pathway, which has been identified as a key regulator of the inducible expression of the antioxidative enzymes NQO1 and HO-1, plays a critical role in cellular defenses against oxidative stress to counteract accumulating ROS in the body [13, 14]. Thus, the expression and activity of the Keap1/Nrf2 signaling pathway were further evaluated in ALX-induced injury MIN6 cells. The injured MIN6 cells exhibited higher Keap1 expression and lower Nrf2 expression, as well as lower expression of HO-1 and NQO1, in whole-cell lysates (Figure 6). In addition, ALX markedly decreased the nuclear Nrf2 protein levels (Figure 6), suggesting that ALX notably suppressed the translocation of Nrf2 from the cytoplasm to the nucleus. These findings revealed that the Keap1/Nrf2 signaling pathway was significantly inhibited in injured MIN6 cells. Then, to confirm the role of the Keap1/Nrf2 signaling pathway in ALX-stimulated injured MIN6 cells, Nrf2 expression was silenced in MIN6 cells by transfection with siNrf2. The results showed that transfection of cells with siNrf2 significantly promoted the ALX-induced decreases in cell viability and proliferation, as well as enhanced the increase in cell apoptosis (Figure 7). Moreover, siNrf2 silencing markedly promoted the ALX induction of intracellular ROS overproduction and DNA damage (Figure 7). Taken together, these in vitro study results demonstrated that the Keap1/Nrf2 signaling pathway participates in the progression of ALX-induced pancreatic *β*-cell injury.

## 5. Conclusion

**Figure 8:**
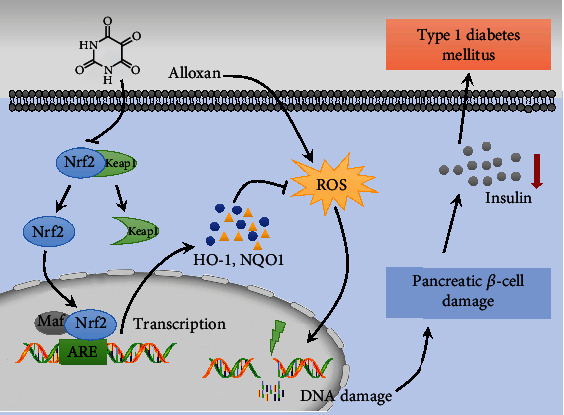
Schematic diagram of mechanism on this research. The Keap1/Nrf2 signaling pathway is closely involved in the pathogenesis and progression of T1DM. The Keap1/Nrf2 signaling pathway could effectively suppress ALX-stimulated intracellular ROS overproduction to protect pancreatic *β*-cell from oxidative stress-induced DNA damage, thereby contributing to the suppression of T1DM development. Conversely, inhibition of the Keap1/Nrf2 signaling pathway significantly promotes the progression of T1DM.

Conclusively, based on the results from these well-controlled in vitro and in vivo studies, it was speculated that the Keap1/Nrf2 signaling pathway is closely involved in the pathogenesis and progression of T1DM. The Keap1/Nrf2 signaling pathway could effectively suppress ALX-stimulated intracellular ROS overproduction to protect pancreatic *β*-cells from oxidative stress-induced DNA damage, thereby contributing to the suppression of T1DM development. Conversely, inhibition of the Keap1/Nrf2 signaling pathway significantly promoted the progression of T1DM (Figure 8). This study indicates that specifically targeting the activation of Keap1/Nrf2 signaling in pancreatic *β*-cells may be a useful pharmacological strategy for the clinical prevention and treatment of T1DM. What factor(s) regulate(s) the Keap1/Nrf2 signaling pathway and how such an understanding can be exploited to control the development of T1DM need to be further defined in future studies.

## Figures and Tables

**Table 1:** Comparison of ALX-stimulated T1DM incidence between WT and Nrf2^−/−^ C57BL/6J mice.

Groups	ALX (mg/kg)	Total mice	T1DM mice	T1DM incidence (%)	**p** value
WT	60	20	15	75	—
Nrf2^−/−^	60	16	16	100	*p* < 0.001

## Data Availability

The datasets generated and/or analyzed during the current study are available from the corresponding author upon reasonable request.

## Supplementary Materials

Supplementary Materials Supplementary Figure 1: the efficiency of Nrf2 silencing. Nrf2 expression was silenced in MIN6 cells by transfection of three Nrf2-specific siRNA (siNrf2-1 or siNrf2-2), respectively. The protein levels of Nrf2 were evaluated by using Western blot analysis. The data shown represent the mean ± SD (*n* = 3). ^∗∗∗^*p* < 0.001 compared with the cells transfected with the control siRNA (siCon).
