# Supplementary material for: Inhibition of the Keap1/Nrf2 Signaling Pathway Significantly Promotes the Progression of Type 1 Diabetes Mellitus
Source: Oxid Med Cell Longev. 2021 Feb 5;2021:7866720. doi: 10.1155/2021/7866720 (PMC7884168; doi:10.1155/2021/7866720)
Supplement: Supplementary Materials — Supplementary Figure 1: the efficiency of Nrf2 silencing. Nrf2 expression was silenced in MIN6 cells by transfection of three Nrf2-specific siRNA (siNrf2-1 or siNrf2-2), respectively. The protein levels of Nrf2 were evaluated by using Western blot analysis. The data shown represent the mean ± SD (n = 3). ∗∗∗p < 0.001 compared with the cells transfected with the control siRNA (siCon). [file 7866720.f1.pptx]

## Slide 1
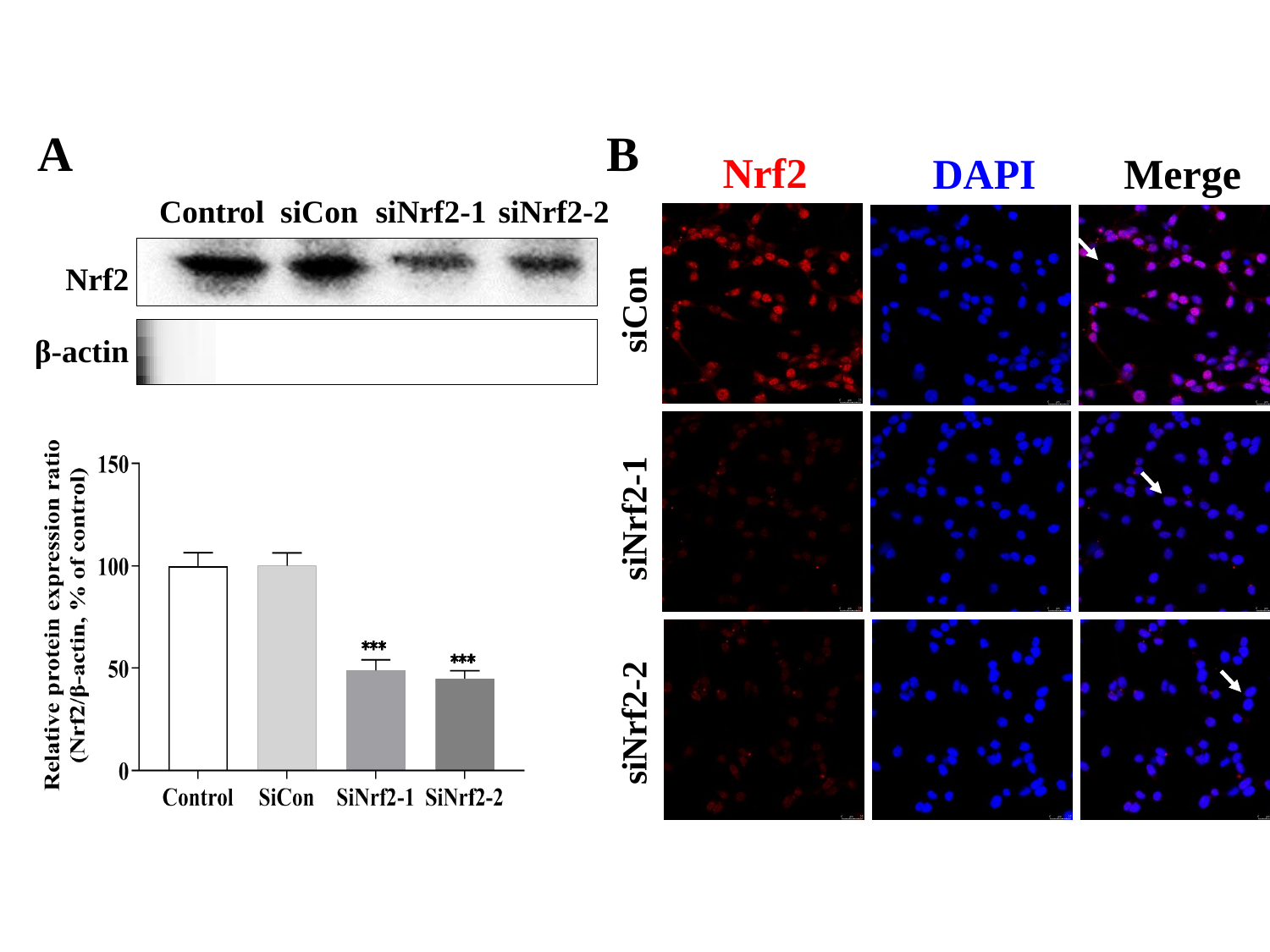

A
B
Nrf2
DAPI
Merge
Control
siCon
siNrf2-1
siNrf2-2
Nrf2
siCon
β-actin
siNrf2-1
siNrf2-2

## Slide 2
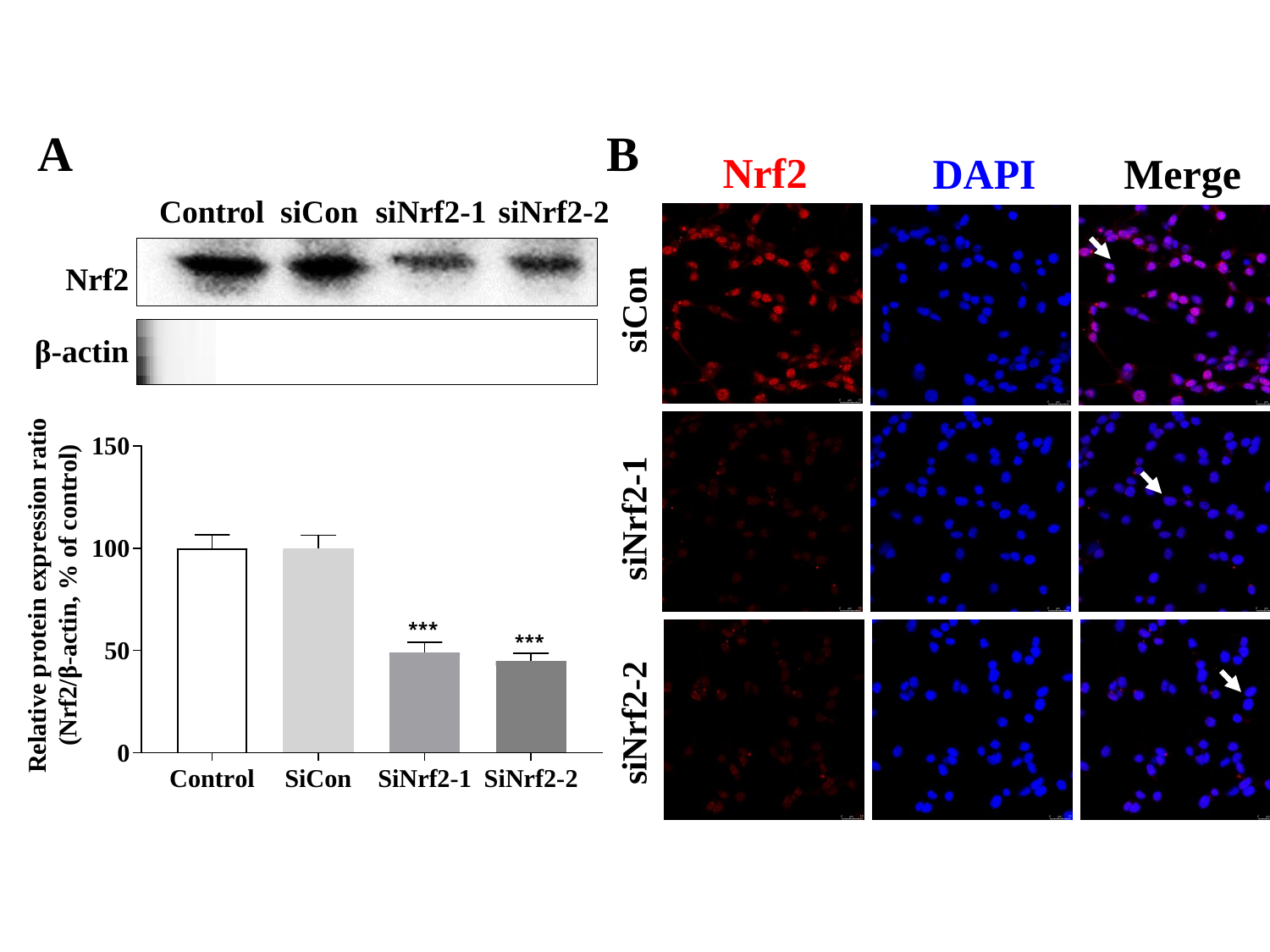

A
B
Nrf2
DAPI
Merge
Control
siCon
siNrf2-1
siNrf2-2
Nrf2
siCon
β-actin
siNrf2-1
siNrf2-2
